# Supplementary material for: Adherence to French and ESGO Quality Indicators in Ovarian Cancer Surgery: An Ad-Hoc Analysis from the Prospective Multicentric CURSOC Study
Source: Cancers (Basel). 2021 Mar 30;13(7):1593. doi: 10.3390/cancers13071593 (PMC8037412; doi:10.3390/cancers13071593)
Supplement: Supplementary file 1 [file cancers-13-01593-s001.pdf]

Supplementary Material: Adherence to French and ESGO Quality Indicators in Ovarian Cancer Surgery: An Ad-Hoc Analysis from the Prospective Multicentric CURSOC Study

Martinez Alejandra, Witold Gertych, Christophe Pomel, Gwenael Ferron, Amelie Lusque, Martina Aida Angeles, Eric Lambaudie, Roman Rouzier, Naoual Bakrin, Francois Golfier, Olivier Glehen, Michel Canis, Nicolas Bourdel, Nicolas Pouget, Pierre-Emmanuel Colombo, Frédéric Guyon, Jacques Meurette and Denis Querleu

Table S1. Quality Indicators evaluated in the study.

Quality Indicators	Evaluated in the Study
Structural indicators	
Indicator 1.1	
20 cytoreductive surgeries performed per center per year	Yes
10 surgical procedures per surgeon per year	Yes
At least 2 surgeons trained to achieve complete cytoreduction	Yes
Indicator 1.2	
Formalized collaboration of the surgical team with a medical oncologist	Yes
Institutional contributions to ovarian cancer clinical trials	Yes
**Process indicators**	
Indicator 2.1	
Preoperative investigations (CT scan, Ca 125)	No*
Indicator 2.2	
Formal pretreatment multidisciplinary approach	No*
Indicator 2.3	
Pre, intra, and postoperative anaesthetic management	No*
Indicator 2.4	
Median laparotomy	No*
Indicator 2.5	
Pelvic and para-aortic lymphadenectomy	No*
Indicator 2.6	
Analytical description of peritoneal lesions and residual disease	No*
Indicator 2.7	
Possibility of frozen section at the time of surgical intervention	Yes
**Outcome indicators**	
Indicator 3.1	
Rate of complete surgical resection either at initial surgery or after neoadjuvant chemotherapy	Yes
Indicator 3.2	
Availability of a morbi-mortality report	Yes

* The analysis per patient of process indicators has not been assessed in this study.
